# Determination of phytonutrients, antioxidant properties and in vitro effect of the microgreen *Trigonella foenum-graecum* L. on H295R carcinoma cells

**DOI:** 10.1007/s13205-025-04578-x

**Published:** 2025-10-22

**Authors:** Tomas Jambor, Lucia Zuscikova, Hana Greifova, Zofia Goc, Agnieszka Gren, Anton Kovacik, Julius Arvay, Norbert Lukac

**Affiliations:** 1 Institute of Applied Biology, Faculty of Biotechnology and Food Sciences, Slovak University of Agriculture in Nitra, Tr. A. Hlinku 2, 949 76 Nitra, Slovakia; 2 Institute of Food Science, Faculty of Biotechnology and Food Sciences, Slovak University of Agriculture in Nitra, Tr. A. Hlinku 2, 949 76 Nitra, Slovakia; 3 Institute of Biology and Earth Sciences, Faculty of Exact and Natural Science, University of the National Education Commission, ul Podchorazych 2, 30-084 Krakow, Poland

**Keywords:** H295R, Microgreens, Hormones, Cytotoxicity, Mitochondrial activity, ROS

## Abstract

This in vitro study quantified the valuable phytochemicals in the *Trigonella foenum-graecum L*. microgreens and determined their antioxidant capacity. In addition, we evaluated the potential effect of *Trigonella* microgreens extract (10–1000 µg/mL) on the human adrenocortical carcinoma cell line (H295R) in terms of morphological and functional parameters such as mitochondrial activity, cell membrane integrity and lysosomal activity after 48 h exposure. Moreover, we determined the potential to generate or inhibit reactive oxygen species tion and changes steroid hormone secretion after respective treatment. The data were collected from three independent experiments performed in triplicate. Collected data passed through Shapiro–Wilk’s normality test, followed by One-way analysis of variance (ANOVA), and Dunnett’s multiple comparison tests.HPLC–DAD analyses revealed rutin, quercetin and p-coumaric acid as the most common phytochemicals present in *Trigonella* microgreens. In addition, DPPH and ABTS assays confirmed a significant potential for scavenging reactive oxygen species, which fluctuated around 191.67 mg TEAC/g d.w. Mitochondrial activity analyses in exposed H295R showed significant inhibition at higher applied doses (300 and 1000 µg/ml). A similar tendency was observed in cell membrane integrity and lysosomal activity. In the case of reactive oxygen species formation, all applied doses significantly inhibited the presented parameter. Interestingly, lower experimental concentrations (150–250 µg/mL) stimulated the release of steroid hormones, however, increasing concentrations caused a progressive decrease in progesterone and testosterone secretion. Nevertheless, the revelation of additional cellular reactions and intracellular mechanisms is certainly necessary for a precise understanding of the effect of microgreens on human health.

## Introduction

Health issues that plague the current population on a large scale not only affect the daily functioning of individuals but also significantly interfere with their socio-economic status. It follows that health is not only a matter of individual concern but also an important social challenge for future generations (Śledzik et al. (2023). The World Health Organization (WHO) plays a key role, coordinating activities and leading global efforts to address health emergencies. Whatever the case, protecting and promoting health should be the primary task of each of us in order to ensure a high-quality and long-life cycle (Jaikumar et al. 2021). A disease state can be defined as any harmful deviation from the normal morphological and functional condition of an organism, directly associated with characteristic symptoms Several of them can be defined as civilization diseases, i.e., diseases that have typical characteristics such as long-lasting jailor persistence, their cause, course and treatment are not clearly defined, they may cause dysfunctions, and they may require rehabilitation or daily care. At the same time, we must emphasize that their increasing development was triggered by civilizational changes, such as increasing industrialization, environmental pollution, stressful conditions, poor physical activity, and poor nutrition (Kitajewska et al. 2014; Barańska and Kłak 2022). If we take a closer look at previous WHO reports and statistics related to the assessment of cancer incidence, there is clear evidence that this civilization disease is one of the leading causes of death worldwide. Although there are recognized some effective therapies to suppress the effects of various types of cancer in humans, the high costs make them inaccessible to common people. In addition, no specific drugs that can definitely suppress malignant growth are known. However, several “alternative” approaches have been described that may improve adverse health conditions (Rezig et al. 2022). Based on these facts, there is constantly growing interest in searching for new medicinal compounds against cancer, which has prompted scientists to look for innovative sources of anticancer compounds in natural sources, including different plant species (Lichota and Gwozdzinski 2018). Over the past decades, many bioactive molecules present in plants such as phenolics, flavonoids, alkaloids and carotenoids have been shown to have the ability to regulate physiological functions and inhibit carcinogenesis in various in vivo and in vitro models (Sharma and Goyal 2015; Bahmani et al. 2016; Iqbal et al. 2017). Recently, growing attention has been directed to microgreens due to their high content of bioactive compounds and favourable nutritional properties. Microgreens are essentially young, immature green plants that are between the sprout and baby greens stages of growth. They have fully established roots, their first true leaves called the cotyledons, are fully developed, and they are harvested later than sprouts. Microgreens contain alkaloids, glycosides, terpenoids, saponins, tannins, and other polyphenols at levels up to 10–100 times higher than in their mature counterparts (Kyriacou et al. 2019; Jambor et al. 2022; Vučetić et al. 2025). Microgreens also have a higher concentration of minerals (K, Ca, Mg, Fe, Mn, Zn, Se) or vitamins, and simultaneously the lower content of anthropogenic contaminants. This is caused by the fact that they are environmentally cultured in regulated environments with no dirt, harmful residues, or contaminated rainwater (Ghoora et al. 2020; Turner et al. 2020). Therefore, there is strong evidence for their several health-promoting properties when consumed regularly (Martinom et al. 2021). Among these plants, *Trigonella foenum-graecum Linn (Fabaceae)* requires special attention. Its phytochemical profile indicates immunomodulatory, antioxidant, hypoglycaemic, and antinociceptive activities, as well as potential antiproliferative effects against certain carcinoma cells (Singh et al. 2016; Thumpati et al. 2025; Mishra et al. 2023). Despite extensive scientific evidence related to *Trigonella* health benefits, the molecular pathways and physiological processes essential for cancer treatment remain insufficiently described. However, it seems that increased concentrations of phenolic compounds, flavonoids, steroidal saponins, alkaloids, etc. present in *Trigonella* microgreens may supports their anticarcinogenic properties (Truzzi et al. 2021; Alu’datt et al. 2024). According to previous in vitro studies (Alsemari et al. 2013; Habib-Martin et al. 2017) cancer cell lines were exposed to Trigonella extract at differing concentrations from 20 to 400 µg/mL, as well as at different time points. Although doses above 100 µg/mL may exceed physiologically relevant levels, their use in in vitro experiments is justified to explore possible mechanisms, benefits, and risks associated with cellular responses. It is difficult to define specific and precise concentrations of Trigonella that can induce physiological changes at an individual level, as bioavailability of the active ingredient, absorption, metabolism and composition of the extract, along with individual physiological processes, are very important factors. Considering published in vitro studies, as well as the essence of our research, we established a robust range of applied doses from 10 to 1000 µg/mL and thus unambiguously determined potential morphological and functional deviations in H295R cells. The aim of the study was to identify the biologically active compounds in *Trigonella* microgreens and to determine their antioxidant potential. In addition, changes in mitochondrial activity, cell membrane integrity, and lysosomal activity of a human adrenocortical carcinoma cell line (NCI-H295R) were assessed in vitro. The potential of the experimental concentrations to trigger oxidative stress and affect steroidogenesis was also determined after 48 h of exposure.

## Material and methods

### Microgreen cultivation, harvesting, processing and extraction

Microgreens *Trigonella foenum-graecum L.,* also known as fenugreek, were germinated from seeds on a specific growth medium in plastic trays, placed in a phytochamber with controlled conditions such as a 16/8 h day/night regime with 22–24 °C and a relative humidity set between 45 and 50%. *Trigonella* microgreen seeds were cultured for 6 days until shoots, roots and pairs of cotyledon leaves appeared. The length of the microgreens before harvesting reached approximately 5–6 cm. Further specific details regarding the growth medium and other cultivation conditions are not disclosed in order to maintain the confidentiality of Microgreens s.r.o. (Bratislava, Slovak Republic), which implemented and supplied the plant material. Samples of freshly harvested microgreens were transported to the AgroBioTech Research Centre (Slovak University of Agriculture in Nitra, Nitra, Slovak Republic) and processed according to established protocols, which include drying at laboratory temperature, mechanical crushing, and weighing in quantities corresponding to the number of analyses (Ivanišová et al. 2020). For extract preparation, one gram of processed *Trigonella* microgreens were extracted by adding 10 mL of 80% (v/v) aqueous ethanol (EtOH; Sigma, St. Louis, MO, USA) for 12 h with constant horizontal shaking at room temperature. Subsequently, the crude extract was centrifuged (9000 rpm, 4 °C, and 5 min), supernatant was collected, filtered through Q-Max RR syringe filter (0.22 µm PVDF, diameter: 25 mm; Frisenette ApS, Denmark), and stored in the dark at 4 °C until following analyses. Further processing and modifications of the crude extract depend on the type of analysis, which are described below.

### High-performance liquid chromatography (HPLC–DAD) analysis

Chromatograph Agilent Infinity 1260 Agilent Technologies GmbH (Agilent Technologies GmbH, Waldbronn, Germany) equipped with a quaternary pump, autosampler and Peltier cooler, column thermostat and DAD detector, was used for the quantification of phenolic compounds in *Trigonella foenum-graecum L.* microgreens extract*.* HPLC separation was performed on LiChroCart 250-4 Purospher reverese phase C18 end-capped column (Merck KGaA, Darmstadt, Germany), and the mobile phases consisted of acetonitrile (A), together with 0.1% phosphoric acid in double-deionized water (v/v) (B). The gradient elution was as follows: 0–1 min isocratic elution (20% A + 80% B), 1–5 min linear gradient elution (25% A + 75% B), 5–15 min linear gradient elution (30% A + 70% B), 15–25 min linear gradient elution (40% A + 60% B). Other specifications were defined as follows: injection volume—3 µL, the flow rate—1 mL/min, post-run equilibration was set up for 3 min, and the column thermostat was heated up to 30 °C while the samples were kept at 4 °C in the sampler manager. The scanning of the spectrum was carried out in the range of 210–410 nm. The spectral data were processed using the Agilent OpenLab Chem Station software for LC RD Systems (Lukšič et al. 2016).

### Total antioxidant capacity

#### DPPH radical scavenging assay

The free radical scavenging ability of *Trigonella* was evaluated using the 2,2-diphenyl-1-picrylhydrazyl (DPPH) method previously performed by Larrauri (Larrauri et al. 1998). A 400 μL of *Trigonella* was added to 3.6 mL of a DPPH working solution prepared by mixing 0.025 g of DPPH (Aldrich, St. Louis, USA) in 100 mL of EtOH (Sigma Aldrich, St. Louis, USA). The absorbance of the mixture was measured using a Jenway 6405 UV/VIS spectrophotometer (Fischer Scientific, Leicestershire, UK) at a wavelength of 515 nm. DPPH inhibition as a percentage of free radical scavenging activity was calculated using the following formula:

$$\% \,{\text{inhibition}} = \left[ {\left( {Ac - As} \right)/\left( {Ac} \right)} \right] \times 100$$

where Ac represents the absorbance of DPPH in solution without *Trigonella* and As represents the absorbance of DPPH in the presence of *Trigonella*. In addition, the free radical scavenging potential was expressed in mg/g Trolox equivalents (TEAC) (Trolox; 6-hydroxy-2,5,7,8-tetramethylchroman-2-carboxylic acid; Sigma-Aldrich, St. Louis, MO, USA).

#### ABTS radical scavenging assay

The procedure of this method was performed according to the previously described protocol for the ABTS (2’-azinobis-(3-ethylbenzothiazoline-6-sulfonic acid; Merck, Darmstadt, Germany) radical scavenging assay by Arnao (Arnao et al. 2001), with slight modification. The cationic radical ABTS• + solution was generated by the reaction between a 2.4 mM potassium persulfate (K2S2O8; Sigma-Aldrich, St. Louis, MO, USA) solution and 7 mM ABTS. The final ABTS* + solution was obtained after a reaction time (approximately 15 h) at room temperature without access to light. Then, the solution was diluted with acetate buffer (0.1 mol/L; pH 4.3) to obtain an absorbance of 0.700 units measured at a wavelength of 734 nm on a spectrophotometer (Shimadzu UV-1800; Cole-Parmer, IL, USA). The absorbance was measured until 20 min after the initial mixing of 50 µL of *Trigonella* extract with 2950 µL of diluted ABTS• + solution, the results were calculated as mg/g trolox equivalents (TEAC) of the sample based on the calibration curve.

### Cancer cell culture

The human adrenocortical carcinoma cell line NCI-H295R was purchased from American Type Culture Collections (ATCC, CRL-2128; Manassas, VA, USA) and cultured according to established protocols and approved in vitro techniques. The initial batch of experimental cell culture was thawed and transferred to 25 cm^2^ plastic culture flasks (TPP AG, Trasadingen, Switzerland) filled with a 1:1 mixture of Dulbecco’s Modified Eagle Medium/Nutrient F-12 Ham (Sigma, St. Louis, MO, USA) supplemented with 1.2 g/L NaHCO3, Molar Hallek, 5 mL BD Nu-Serum (BD Bioscience, Bath, UK), and 5 mL/L ITSC Premix (Corning, AZ, USA), where the cells were grown and subcultured for at least three additional passages to achieve optimal physiological state. Cells were maintained in a humidified atmosphere of 95% air, 5% CO_2_ and 37 °C in a CO_2_ incubator. To avoid microbial contamination H295R cells were regularly screened by the PlasmoTestTM (InvivoGen Inc, San Diego, CA, USA), and they were mycoplasma-free.

### In vitro treatment

The experimental model of H295R cells was routinely passaged after reaching 75–80% confluency, and experimental load was realised between 8 and 25th passages. In brief, after regular subculturing, cells were seeded at a density of 30,000 cells/cm^2^ on sterile 96-well plates and pre-cultured for 24 h in CO_2_ incubator with defined conditions described previously. Meanwhile, prepared crude extract of *Trigonella* microgreens was subjected to evaporation (Stuart RE300DB rotary evaporator, Bibby Scientific Limited Inc, UK) under reduced pressure (vacuum pump KNF N838.1.2KT.45.18, Freiburg, Germany) at 40 °C in order to remove any residual EtOH. Afterwards, the extract was dissolved in DMSO (Sigma, St. Louis, MO, USA), adjusted to 2000 µg/mL, and diluted in cell culture media to the final concentrations: 10; 50; 100; 150; 200; 250; 300; and 1000 µg/mL. Subsequently, H295R cells were incubated for 48 h in vitro. The experimental concentration range of *Trigonella* microgreens was set up based on our pilot range-finding study (Jambor et al. 2021). The concentration of DMSO solvent did not exceed 0.6% (v/v)., It was also included on each experimental plate as a negative control. Each experiment was conducted at least three times, using cells from different passages.

### Mitochondrial activity assay

Mitochondrial activity of H295R cells exposed to different doses of *Trigonella* microgreen extract was assessed using MTT (3–4,5-dimethylthiazol-2-yl)−2,5-diphenyltetrazolium bromide; Sigma-Aldrich, St. Louis, MO, USA) assay. This method essentially assesses the reduction of a yellow tetrazolium salt to purple formazan crystals by means of functionally active mitochondrial dehydrogenase enzyme action (Mosmann 1983). Briefly, treated cells seeded at the density of 30,000/cm^2^ were stained with MTT working solution consisting of tetrazolium salt dissolved in cell culture medium (1:1) for 2 h, and keept in CO_2_ incubator. Afterward, formed formazan crystals were dissolved in isopropanol (p.a. CentralChem, Bratislava, Slovak Republic) and the optical density was measured with a MultiScan FC ELISA reader (ThermoFisher Scientific, Vantaa, Finland) with a wavelength set at 570–620 nm. The obtained data were expressed as a percentage of the control group (i.e., the optical density of untreated cells).

### Cytotoxic assays

The potential of *Trigonella* to induce cytotoxicity was evaluated by 5-carboxyfluorescein diacetate, acetoxymethyl ester (CFDA-AM; Thermo Fisher Scientific, Waltham, Massachusetts, USA) assay, and neutral red uptake (NR; Thermo Fisher Scientific, Waltham, Massachusetts, USA). These methods combine the evaluation of esterase activity and cell membrane integrity (CFDA-AM; (Schreer et al. 2005), while NRU determines the uptake and lysosomal retention of neutral red dye (Yawer et al. 2022) in exposed H295R cells. In brief, exposed cells (seeding density 30,000/cm^2^) were incubated with CFDA-AM (final concentration: 4 µM) dissolved in cell culture media of 0.5 h followed by fluorescence reading (excitation/emission: 485/530 nm) carried out by microplate reader GloMax^®^-Multi + (Promega Corporation, Madison, USA). Then, CFDA-AM was removed, cells were washed, and NR was added (0.005% w/v in cell culture media). After 2.5 h incubation, cells were lysed by 1% (v/v) acetic acid (CentralChem, Bratislava, Slovak Republic) in 50% (v/v) EtOH. The absorbance was measured at 525/690 nm wavelength by the microplate reader GloMax^®^-Multi +. All data obtained were expressed as a percentage of the control group (i.e., the optical density of untreated cells).

### Intracellular reactive oxygens species (ROS) production

Quantification of ROS generation was assessed by a chemiluminescence assay using luminol (5-amino-2,3-dihydro-1,4-phthalazinedione; Sigma-Aldrich, St. Louis, USA) as a probe (Kashou et al. 2013), with slight modification. Briefly, exposed cells were incubated with 12.5 μL of luminol working solution (5 mmol/L), while negative control wells contained 200 μL of Dulbecco’s phosphate-buffered saline (DPBS; Sigma-Aldrich, St. Louis, USA) and 12.5 μL of luminol working solution. Positive control wells consisted of 200 μL of DPBS with 62.5 μL of 30% hydrogen peroxide (H202; Sigma-Aldrich, St. Louis, USA) and 12.5 μL of working solution. Chemiluminescence was read in 24-well plates every 1 min in 15 cycles. A combined spectro-fluoro luminometer (GloMax^®^—Multi + Microplate Multimode reader with Instinct^®^; Promega Corporation, Madison, USA) was used to measure relative light units/s (RLU/s).

### Steroid hormone secretion

Quantification of sex steroid hormones secretion was determined by enzyme-linked immunosorbent assay (ELISA). The concentrations of progesterone (cat. no. K00225, Dialab, Austria) and testosterone (cat. no. K00234, Dialab, Austria) were measured with commercial kits directly from H295R culture medium after 48 h of *Trigonella* microgreens treatment. The procedure of hormone determination was performed according to the kit instructions for use. Absorbance was measured with an ELISA reader (Multiscan FC, ThermoFisher Scientific, Vantaa, Finland) with the appropriate absorbance set at 450 nm. All data obtained were expressed as a percentage of the control group (i.e. optical density of untreated cells). The intra- and inter- assay coefficients of variability of progesterone were estimated as ≤ 4.0% (intra-variability) and ≤ 9.3% (inter-variability) with 0.05 ng/mL sensitivity while, for testosterone were established as ≤ 7.0% (intra-variability) and ≤ 8.3% (inter-variability) with 0.10 ng/mL sensitivity.

### Statistical analyses

Statistical analyses were carried out by using the GraphPad Prism program (version 6.07, GraphPad Software, Inc., Sand Diego, CA, USA). Data representing independently repeated experiments (at least three independent repetitions, unless stated otherwise) were combined and used for further analysis. Collected data passed through Shapiro–Wilk’s normality test, followed by assessing of descriptive statistical characteristic (mean, standard error and standard deviation). One-way analysis of variance (ANOVA), followed by Dunnett’s multiple comparison tests, was used to examine differences between the experimental and control groups. The results were expressed as the mean ± standard error meaning (SEM). P-values equal to or lower than 0.05 were considered statistically significant.

## Results

### Prevalence of phenolic compounds in Trigonella microgreen

HPLC–DAD analyses revealed presence of different phenolic compound summarized in Table 1. The most prevalent flavonoids were rutin and quercetin, while the myricetin or resveratrol were detected in lower value. On the other hand, a significant level of phenolic precursors such as p-coumaric acid or 4-OH benzoic acid were clearly detected in *Trigonella* microgreens. Other phenolic derivates such as caffeic acid, ferulic acid, and trans-cinnamic acid were detected, but not in significant level. These results from HPLC–DAD analyses confirmed extremely high concentrations of some flavonoids, especially rutin with 496.26 ± 12.55 mg/kg.

**Table 1:** Major phenolic compounds identified and quantified (mg/kg) in *Trigonella foenum-graecum L.* microgreen by HPLC–DAD analyses

Phenolic compounds	Concentration (mg/kg d.w.)
4-OH benzoic acid	8.26 (± 0.058)
Caffeic acid	14.66 (± 0.074)
p-coumaric acid	48.58 (± 0.662)
Rutin	496.26 (± 4.292)
Ferulic acid	7.30 (± 0.035)
Myricetin	2.94 (± 0.039)
Resveratrol	1.81 (± 0.029)
Quercetin	11.54 (± 0.457)
Trans-cinnamic acid	3.42 (± 0.020)

Data are presented as means (± SEM) from three independent measurements

*d. w.* dry weight

### Free-radical scavenging activity of Trigonella microgreen

The antioxidant activity of experimental extract was evaluated by two different methods, when DPPH assay established free-radical scavenging activity at 9.12 ± 0.03 mg TEAC/g d.w., and ABTS method revealed the scavenging potential at 191.67 ± 1.47 mg TEAC/g d.w. Both parameters are summarized in Table 2.

**Table 2:** Free-radical scavenging activity of *Trigonella* microgreen evaluated by DPPH and ABTS assays

Parameter	Value
DPPH assay	9.12 (± 0.030) mg TEAC/g
ABTS assay	191.67 (± 1.471) mg TEAC/g

Data are presented as means (± SEM) from three independent measurements

*d. w.* dry weight, *TEAC* trolox equivalents

### Mitochondrial activity of treated H295R cells

**Fig. 1:**
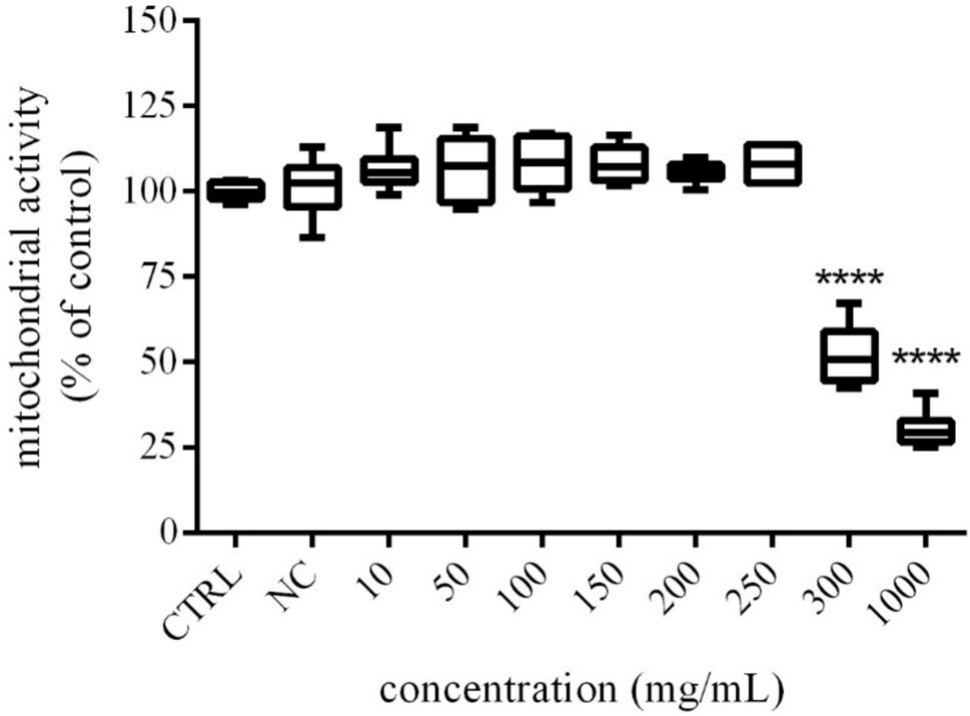
The effects of *Trigonella foenum-graecum* L. on H295R cells mitochondrial activity after 48 h exposure in vitro. *CTRL* control group (untreated cells), *NC* negative control (0.6% DMSO). The data are presented as means (± SEM) optical density percent of the control (untreated) and experimental extract´s treated groups. The data were collected from three independent experiments performed in triplicates. Levels of significance were established at ****(p < 0.0001); ***(p < 0.001), and **(p < 0.01). Statistical differences between the values of control and treated groups are indicated by an asterisk (*)

The mitochondrial activity of exposed H295R cells was estimated using MTT assay, and the results revealed dose- dependent effects (Fig. 1). Higher concentrations of *Trigonella foenum-graecum L*. (300 and 1000 µg/mL) displayed decreased mitochondrial activity of cells with significant (*p* < 0.0001) changes (52.18 ± 3.72% vs. 30.44 ± 2.23%) compared to the control (untreated) cells (100.00 ± 1.06%) after 48 h exposure. Regarding the effects of lower tested concentrations, a significant elevation or depression of mitochondrial activity was not observed in samples corresponding to 150, 200 and 250 µg/mL concentrations (106.10 ± 2.18%; 105.8 ± 1.26%; 106.20 ± 1.75%), in comparison to the control group.

### Cytotoxic effect of Trigonella microgreen

**Fig. 2:**
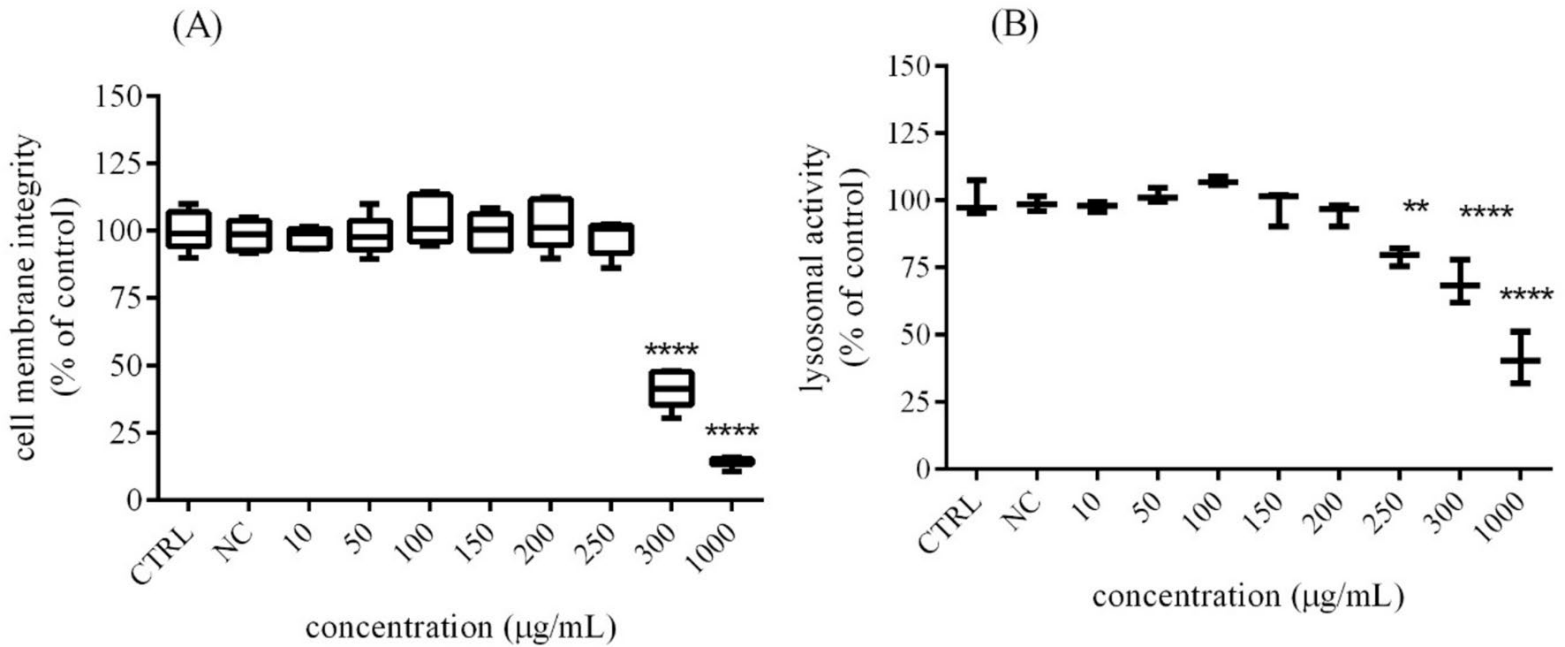
The cytotoxic effects of *Trigonella foenum-graecum* L. on H295R cells activity after 48 h exposure in vitro. *CTRL* control group (untreated cells), *NC* negative control (0.6% DMSO). The data are presented as means (± SEM) optical density percent of the control (untreated) and experimental extract´s treated groups. The data were collected from three independent experiments performed in triplicates. Levels of significance were established at ****(p < 0.0001), and **(p < 0.01). Statistical differences between the values of control and treated groups are indicated by an asterisk (*)

The cytotoxic effects were evaluated using CFDA-AM assay and NR uptake. The results confirmed that experimental concentrations of *Trigonella foenum-graecum L*. equal 300 µg/mL led to a significant (*p* < 0.0001) decrease of cell membrane integrity (41.03 ± 2.79%) compared to the untreated (control) cells (100.00 ± 2.93%). While an even more visible effect (*p* < 0.0001) was observed in the cells treated with the highest concentration 1000 µg/mL (14.29 ± 0.73%) (Fig. 2A). Similar tendency was confirmed by NRU, when the lower applied doses (up to 200 µg/mL) did not have cytotoxic effect. Overleaf, increasing doses of Trigonella (250 µg/mL and 300 µg/mL) caused significant cytotoxicity after 48 h exposure compared to the control group (100.00 ± 3.78%). Results from cytotoxic evaluation are summarized in Fig. 2B.

### Assessment of ROS production

**Fig. 3:**
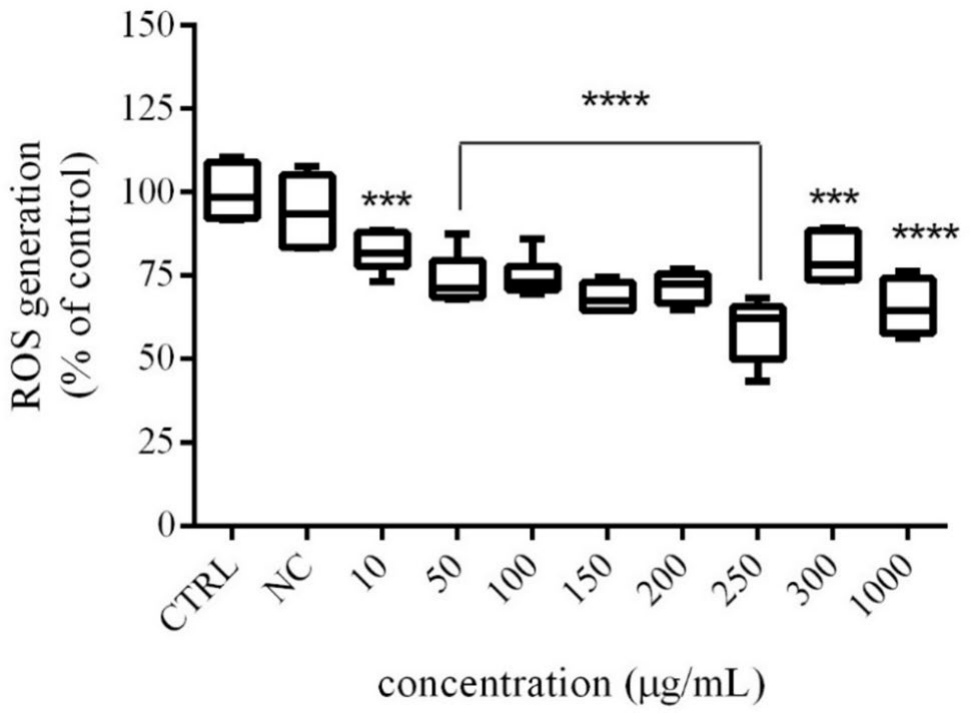
The effects of *Trigonella foenum-graecum* L. on ROS generation in H295R cells activity after 48 h exposure in vitro. *CTRL* control group (untreated cells), *NC* negative control (0.6% DMSO). The data are presented as means (± SEM) optical density percent of the control (untreated) and experimental extract´s treated groups. The data were collected from three independent experiments performed in triplicates. Levels of significance were established at ****(p < 0.0001), and ***(p < 0.001). Statistical differences between the values of control and treated groups are indicated by an asterisk (*)

The intracellular formation of ROS was evaluated by chemiluminescence method using luminol as a probe. A significant (*p* < 0.0001; *p* < 0.001) inhibition of ROS production was confirmed after 48 h exposure to *Trigonella foenum-graecum L*. in all experimental doses (Fig. 3). The value decreased (*p* < 0.001) by approx. 19% in comparison to control (100.00 ± 3.34%) in samples treated 10 µg/mL and 300 µg/mL of microgreen extract (82.08 ± 2.37%; 80.17 ± 2.98%). While in the remaining samples the significant (*p* < 0.0001) decrease was approx. 29%. However, the most effective (*p* < 0.0001) inhibition of ROS production was noted in samples treated with a concentration of 250 µg/mL (58.85 ± 3.84%).

### Assessment of steroid hormone secretion

**Fig. 4:**
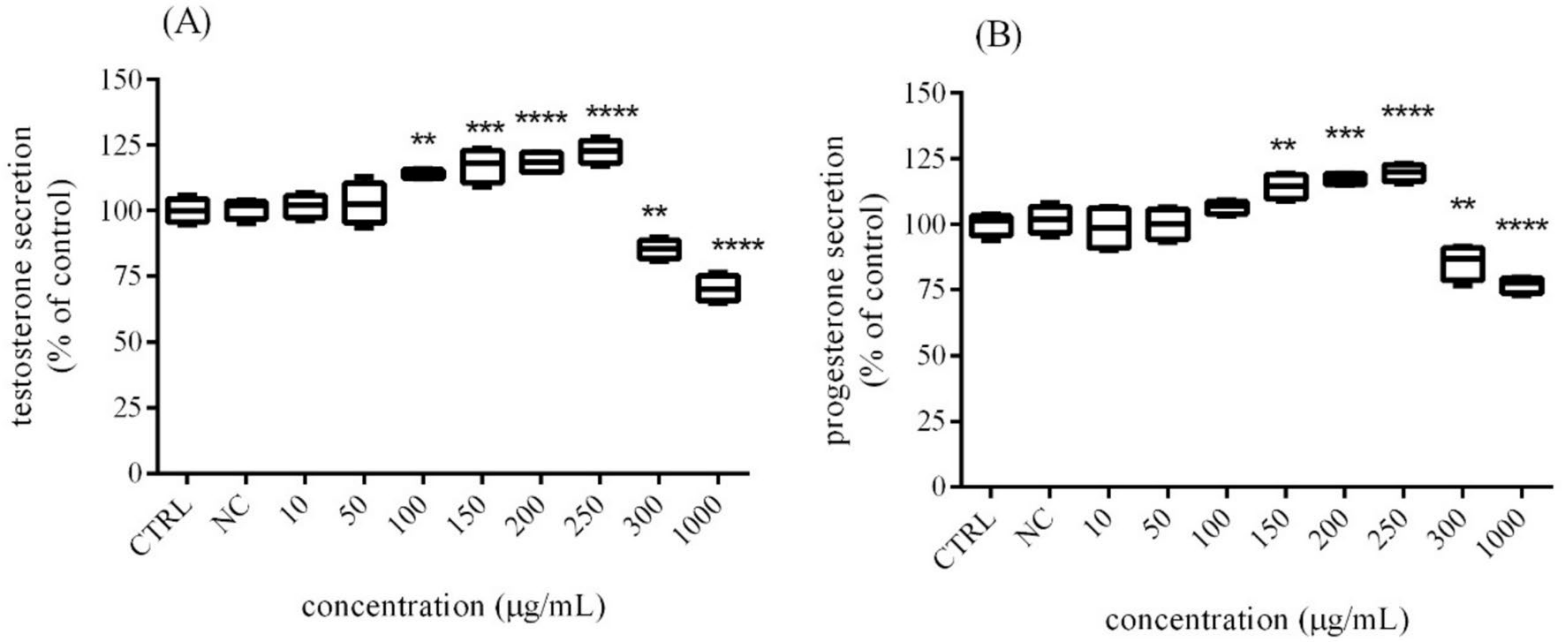
The effects of *Trigonella foenum-graecum* L. on **A** testosterone and **B** progesterone release by H295R cells after 48 h exposure in vitro. *CTRL* control group (untreated cells), *NC* negative control (0.6% DMSO). The data are presented as means (± SEM) optical density percent of the control (untreated) and experimental extract´s treated groups. The data were collected from three independent experiments performed in triplicates. Levels of significance were established at ****(p < 0.0001), and **(p < 0.01). Statistical differences between the values of control and treated groups are indicated by an asterisk (*)

The effects of *Trigonella foenum-graecum L.* on H295R cell production of sex-steroid hormones was evaluated by ELISA method. In the case of testosterone, a significant (*p* < 0.01) stimulation was recorded at 100 µg/mL (114.07 ± 0.81%), followed by significant (*p* < 0.0001; *p* < 0.001) growth at 150 µg/mL, 200 µg/mL and 250 µg/mL of experimental extract (Fig. 4A). Similar effect was confirmed in case of progesterone, when significant (*p* < 0.0001; *p* < 0.001;* p* < 0.01) stimulation starting at 150 µg/mL (114.30 ± 2.35%) until 250 µg/mL (119.60 ± 1.63%) compared to the control group (100.00 ± 2.14%). Inversely the highest doses, 300 µg/mL and 1000 μg/mL of microgreen extract caused a significant (*p* < 0.01; *p* < 0.0001) decline in testosterone (85.21 ± 1.88%; 70.36 ± 2.46%;) secretion compared to the control cells (100.00 ± 2.29%), as well as in progesterone (85.40 ± 3.29%; 76.96 ± 1.56%;) release compared to the control (untreated) cells (Fig. 4B).

## Discussion

**Fig. 5:**
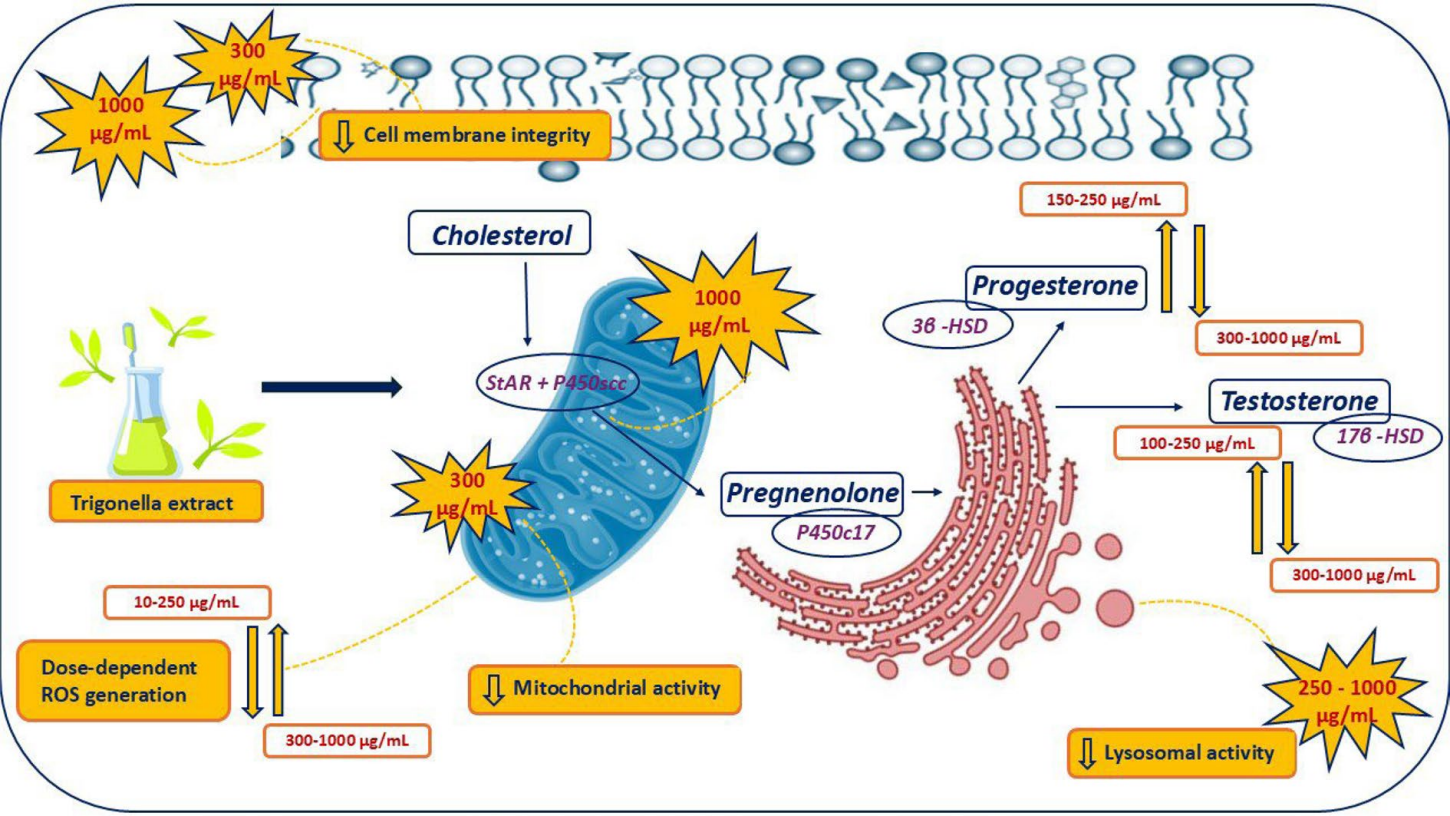
Graphical summary of the major findings

The relationship between plant-based diets and cancer has been investigated in many studies, and various scientific evidence suggests a significant role for various plants in cancer prevention. Currently, increasing attention is being focused worldwide on microgreens due to their rich phytochemical profiles and ability to stimulate physiological functions at both the cellular and systemic levels. Trigonella foenum-graecum L. grown as a microgreen is characterized by its antidiabetic, antioxidant, gastroprotective, hepatoprotective and anticancer properties with a high diversity of phytochemicals. (Raiola et al. 2017; Ren et al. 2024). Our results revealed a diverse content of phytochemicals, with flavonoids such as rutin or quercetin being the most prevalent. Ghevariya (Ghevariya et al. 2023) also confirmed higher total phenolics (TP) and flavonoids (TF) content in Trigonella microgreens compared to seeds or stem and leaf of the adult plant. The highest TP was determined to be approximately 64 mg/GAE d.w., while TF fluctuated at 19 mg/QCE d.w. While similar levels of TP or TF were found in Trigonella seeds, on the contrary, adult plant stems and leaves did not reach comparable concentrations. Kumar (Kumar et al. 2024) used HPLC–DAD method relevant for the identification of phytochemicals in Trigonella microgreens. The results of this study showed significantly increased concentrations of p-coumaric acid (5.83 mg/g), ferulic acid (3.92 mg/g) and resveratrol (2.12 mg/g), in agreement with our findings. On the other hand, the most abundant phytochemical (rutin) detected in our study was not present at high levels in Kumar’s study. Ghevariya (Ghevariya et al. 2023) evaluated the total antioxidant content (TAC) using DPPH assay, finding Trigonella microgreens exhibited the highest scavenging activity, followed by seeds, leaves and stems of adult plants. TAC of Trigonella was also investigated using DPPH assay and FRAP activity in a previous study (Ahmad et al. 2022). The results obtained indicate an excellent potential to inhibit ROS generation, especially at 100 µM Trigonella extract. Although the diverse biochemical profile and strong antioxidant properties of Trigonella microgreens have been confirmed, experimental studies focusing on basic cellular parameters and molecular pathways relevant to cancer mechanisms remain limited. Mitochondrial activity of H295R cells treated with Trigonella microgreens was assessed by MTT assay and our results confirmed significant inhibition at the highest experimental doses (300 and 100 µg/mL). To comprehensively evaluate the experimental extract´s effect, it was necessary to reveal possible changes in cell membrane integrity and lysosomal activity of exposed cells. At the same doses, both parameters were significantly affected. The potential to inhibit cell proliferation induced by Trigonella foenum-graecum L. was evaluated in a previous study (Khalil et al. 2015). Human liver cells HepG2 were exposed to different experimental concentrations (50–2000 µg/mL) of Trigonella extract for 48 h. The mitochondrial was evaluated by MTT assay. The results showed a significant dose-dependent cytotoxic effects on HepG2 cell line. A significant decrease of cell viability was observed at 100 μg/mL and higher concentrations of *Trigonella* compared to untreated cells. At the highest dose (1000 μg/mL), cells growth stopped, cell adhesion was disrupted, and HepG2 cells became non-viable. Furthermore, higher doses of the extract, starting from 100 µg/mL, affected cell morphology (smaller, shrunken and rounded) and significantly increased caspase-3 activity, which plays a key role in the apoptosis process. The cytotoxic effect of Trigonella foenum-graecum L. extract with different concentrations (100 μg/mL, 500 μg/mL, 750 μg/mL, 1000 μg/mL, 1500 μg/mL) was confirmed for SH-SY5Y neuroblastoma cells after 24 h cultivation in vitro (Ürkmez et al. 2022). The results revealed a dose-dependent cytotoxicity of *Trigonella* extract. The highest dose (750 µg/mL) progressively inhibited mitochondrial activity evaluated by the MTT assay. In addition, *Trigonella* treatments caused significant inhibition of cell proliferation, cell migration, and progressively affect cell morphology. Moreover, in cells exposed to higher experimental doses of *Trigonella*, chromosomal DNA was fragmented into long internucleosomal fragments. Compared to our results, the discussed studies confirmed the hypothesis of dose-dependent effects on metabolic activity of exposed cells, accompanied by significant inhibition of carcinoma cells proliferation at higher applied doses of *Trigonella* (Alrumaihi et al. 2021; Khoja et al. 2022). Oxidative stress is closely related to the occurrence of common human diseases including cancer (Klaunig et al. 2011; Bhat et al. 2015). On the other hand, antioxidants have become an important strategy in scavenging ROS and thus in cancer chemoprevention. Therefore, the various phytochemicals detected in our experimental microgreen *Trigonella foenum-graecum L*. may have an important effect on intracellular pathways of exposed cells, and on physiological processes in general (Roleira et al. 2015; Mahbub et al. 2015). Results of our study confirmed a significant inhibition of ROS generation in H295R cells treated by experimental extract from *Trigonella* microgreen. A study by (Jayaraman and Ramasamy 2024) demonstrated the potential of *Trigonella* microgreens as a source of nutraceutical compounds with promising antioxidant properties, confirmed by ABTS and TAC assays. Our results align with these findings, showing that even lower concentrations of the microgreen *Trigonella foenum-graecum L.* possess significant antioxidant potential, and progressively reduce superoxide radical production in H295R adrenocortical carcinoma cells. In addition, study of Kaviarasan (Kaviarasan and Anuradha 2007) observed, that *Trigonella* extract effectively suppress OH· radicals in rat liver mitochondria. A recent in vivo study showed that *Trigonella foenum-graecum L*. ameliorative effects oxidative stress-mediated liver damage in rats (Mayakrishnan et al. 2015). Different markers such as lipid peroxidation, reduced glutathione (GSH), superoxide dismutase (SOD), catalase (CAT), and glutathione peroxidase (GPx) were analyzed in liver tissue homogenate. Tha experimental data revealed that the applied extract had a beneficial effect related to the decrease lipid peroxidation level, reduced significantly the expression of liver endoplasmic reticulum stress biomarkers, and increased hepatic antioxidants. Various cell lines cultured in vitro serve as ideal models to study the biological effects of plant extracts and various phytochemicals, enabling the detection of physiological changes resulting from inter- or intracellular changes (Sharma et al. 2024; Xavier et al. 2024). The capacity of microgreens to influence steroidogenic pathways and the mechanism by which these plants may interfere with the function of steroidogenic enzymes remain relatively unexplored. Our in vitro study demonstrates the effect of different concentrations of *Trigonella foenum-graecum L*. on steroidogenesis in the human H295R cell line (specifically progesterone and testosterone secretion). Different in vitro and in vivo studies indicated that *Trigonella* extracts may affect testosterone secretion through several mechanisms. Glycoside-rich fraction of *Trigonella foenum-graecum L*. seeds such as saponins and sapogenins have shown androgenic and anabolic activity (Wankhede et al. 2016). In this present study, *Trigonella* supplementation (300 mg twice a day) cause significant increase of free testosterone without reduction in total testosterone in male subjects during 8 weeks of resistance training program. Another study evaluated the efficacy of *Trigonella* seed extract (600 mg/day for 12 weeks) on androgen deficiency, sexual function, and serum androgen concentration in healthy aging men. Serum total testosterone and free testosterone were increased compared to placebo (Rao et al. 2016). These results are supported by further in vivo studies in rats. Rats with streptozotocin-induced diabetes were treated with 300 mg/kg aqueous *Trigonella* seed extract. The results indicated that after 4 weeks of *Trigonella* treatment, there was improvement in blood glucose levels, lipid profile, liver and kidney function. Similarly, Al-Chalabi (Al-Chalabi et al. 2019) confirmed stimulation and increase in luteinizing hormone and testosterone levels. In another study, it was observed that administration of lower levels of *Trigonella* extract (35 mg/kg) to male rats for 28 days increased serum testosterone levels and did not affect normal testicular architecture (Aswar et al. 2024). Conversely, (Singh et al. 2022) demonstrated that administration of *Trigonella* extract (600 mg/kg body weight/day) after 28 and 56 days can negatively affect the morphology of the testis epididymis and seminal vesicles. These disorders subsequently inhibit testosterone secretion, followed by a decrease in the activity of steroidogenic enzymes such as 3β-HSD and 17β-HSD. In turn, other studies aimed at evaluating the possible effects of different dosage forms of *Trigonella* seeds on the male reproductive system in animals (Badry et al. 2021). In this study, male albino rats were treated by the administration of either powder (200 mg/kg), aqueous (500 mg/mL) or oily extract (200 mg/mL) forms of *Trigonella* for 8 weeks. Progesterone level was remarkably elevated in the oily form, followed by the powder and aqueous form. In case of testosterone levels were detected higher solely in the aqueous form. The effect of the aqueous form on the male hormonal levels has been significantly noticed with remarkable changes in the sperm vitality as well the sperm count. Instead, the oily form showed a devastating action on all the evaluated parameters. According to current studies, it can be stated that the results obtained can be influenced by various factors that should be considered when comparing conclusions: the age of the animal, the concentration and form of the *Trigonella* extract, the time of treatment, and others. Figure 5 summarize key findings of our study.

## Conclusion

Our in vitro study on the potential effect of *Trigonella* microgreens on H295R cells revealed a unique phytochemical profile with excellent antioxidant properties. The observed intracellular changes in mitochondrial activity and cell membrane integrity at higher experimental doses could indicate inhibition of carcinogenic processes. In addition, lower doses of the microgreen extract applied, can stimulate steroid hormone secretion and maintain endocrine balance in H295R cells.

## Data Availability

The datasets generated during and/or analysed in this study are available from the corresponding author upon reasonable request.
